# A single immunization of *Borreliella burgdorferi*-infected mice with Vanguard crLyme elicits robust antibody responses to diverse strains and variants of outer surface protein C

**DOI:** 10.1128/iai.00396-24

**Published:** 2024-10-22

**Authors:** Gavin Z. Chambers, Kathryn M. F. Chambers, Richard T. Marconi

**Affiliations:** 1 Department of Microbiology and Immunology, Virginia Commonwealth University Medical Center, Richmond, Virginia, USA; University of California Davis, Davis, California, USA

**Keywords:** CH14, crLyme, DCFL4, dogs, Lyme disease, Lyme vaccines, OspA, OspC

## Abstract

Lyme disease, caused by *Borreliella burgdorferi* and related species*,* is a growing health threat to companion animals across North America and Europe. Vaccination is an important preventive tool used widely in dogs living in, or near, endemic regions. In this report, we assessed anti-outer surface protein (Osp) A and anti-OspC antibody responses in *B. burgdorferi*-infected and -naïve mice (C3H/HeN) after immunization with a murine-optimized single dose of the Lyme disease subunit vaccine, Vanguard crLyme. crLyme is comprised of OspA and an OspC chimeritope-based immunogen designated as CH14. Mice that were infected and immunized developed higher levels of anti-OspC antibodies (Abs) than those infected only or that received one vaccine dose. The anti-OspC Abs that developed in the infected/immunized mice bound to all OspC variants tested (*n* = 22), whereas OspC Abs in serum from infected mice bound predominantly to the OspC variant (type A) produced by the infecting *B. burgdorferi* strain. Consistent with the absence of OspA expression in infected mammals, none of the infected mice developed Abs to OspA and did not develop anti-OspA Abs after single dose immunization. Lastly, serum from infected/immunized mice displayed significantly higher and broader killing activity than serum from non-immunized infected mice. The results of this study demonstrate that a single vaccination of actively infected mice results in strong anti-OspC Ab responses. This study contributes to our understanding of Ab responses to vaccination in actively infected mammals.

## INTRODUCTION

*Borreliella burgdorferi*, the primary causative agent of Lyme disease in North America (1, 2), is the most common tick-borne pathogen in the northern hemisphere (3, 4). The American College of Veterinary Internal Medicine recommends that dogs residing in, or near, endemic regions for Lyme disease be screened yearly for exposure to tick-borne pathogens (5). In 2023, the Companion Animal Parasite Council (CAPC) reported that 465,700 dogs in the United States tested positive for antibodies (Abs) to *B. burgdorferi*. The actual number of positive tests is much higher as only about 1/3 of test results are cataloged by CAPC. Antibiotic therapy generally results in positive clinical outcomes when canine Lyme disease is diagnosed and treated early (5). However, if untreated, transient intermittent lameness, polyarthritis, and potentially fatal nephritis may develop.

Preventive strategies for tick-borne diseases in dogs include using parasiticides and, in the case of Lyme disease, vaccination (6). The composition and differences among commercially available canine Lyme disease vaccines are reviewed in ref. (6). Vanguard crLyme (Zoetis Petcare), the focus of this study, is a subunit vaccine for preventing *B. burgdorferi* infection in dogs (7, 8). crLyme consists of two recombinant proteins: OspA and an OspC-based chimeric epitope protein (chimeritope) designated as CH14 (7). crLyme is designed to elicit focused Ab responses against OspA (9) and OspC (10, 11). OspA, an adhesin that binds to the *Ixodes scapularis* TROSPA receptor (12), is selectively expressed in ticks (13). In contrast, OspC, which is required for transmission from ticks to mammals (14, 15), is among the most highly expressed *Borreliella* proteins during early infection of mammals (16).

Worldwide, seven different variants of OspA have been delineated based on reactivity with monoclonal Abs (17). In contrast, more than 35 distinct OspC variants, commonly called OspC types (18, 19), have been identified. Ab responses to OspC proteins are largely type-specific (20). The epitope-containing domains (ECDs) responsible for the OspC type-specific Ab responses have been identified. The L5 and H5 ECDs span residues 136–150 and 168–203 (numbering based on the *B. burgdorferi* B31 OspC), respectively (19, 21, 22). Consistent with the sequence diversity of the L5 and H5 ECDs, a single OspC-type protein does not provide broad protective immunity against strains expressing different OspC types (23). To design an OspC-based immunogen with broad protective capability, OspC-based chimeric epitope proteins (chimeritopes) comprised of L5 and H5 ECDs from diverse OspC types have been generated and tested (7, 22, 24, 25). In a head-to-head study in canines, the CH14 chimeritope immunogen in crLyme (Zoetis) elicited more broadly reactive anti-OspC Ab responses than other commercially available canine Lyme disease vaccines (7, 26). crLyme is administered in a two-dose series delivered approximately 3 weeks apart during the first year of use, with yearly boosters recommended.

In this report, we delivered a mouse-optimized dose of crLyme to *B. burgdorferi* (strain B31; OspC type A; OspA type 1)-infected and -naïve mice to determine whether immunologically primed mice develop higher IgG titers to vaccination. In addition, we compared the breadth of the Ab response elicited to 22 diverse OspC variants and measured the bactericidal activity of serum from each study group. The data presented provide insight into how infected animals may respond to single-dose vaccination crLyme.

## MATERIALS AND METHODS

### Bacterial cultivation

Clonal populations of isolates used in this study were obtained by subsurface plating (27). *B. burgdorferi* strain B31 (OspC type A) was originally recovered from an *I. scapularis* tick (New York, USA*). B. burgdorferi* strains DRI85A*,* DRI16C, and DRI03C (OspC types DRI85A, E, and H, respectively) were recovered from infected dogs (28). All clones were cultivated at 34°C in BSK-H medium supplemented with 6% rabbit serum (Sigma). Growth was monitored by examination of wet mounts using dark-field microscopy.

### Generation of recombinant proteins

Genes encoding OspA, VlsE, 22 different OspC types, and the chimeric proteins CH14 (7) and DCFL4 were codon-optimized, synthesized, and provided by the supplier (GenScript) in pET45b(+). DCFL4 encodes a novel chimeric diagnostic antigen comprised of immunodominant domains of four *B. burgdorferi* proteins (29). All protein production and purification methods were as previously described (29). Protein concentrations were determined by bicinchoninic acid assay (Pierce), and protein purity was assessed by SDS-PAGE using Anykda precast gels (Bio-Rad) and standard methods.

### Infection of mice with increasing numbers of *B. burgdorferi* B31

To determine the optimal infectious dose of *B. burgdorferi,* C3H/HeN mice (Charles River) were infected (five mice per group) by subcutaneous needle inoculation with *B. burgdorferi* strain B31 cells using a dose range of 10^3^–10^6^ cells. The mice were euthanized 21 days after inoculation by CO_2_ asphyxiation. Blood was collected by cardiac puncture, and the serum was harvested and screened by enzyme-linked immunosorbent assays (ELISA) for Abs to OspA, CH14, VlsE, and DCFL4. For all subsequent analyses, a dose of 10^4^ cells was used to infect the mice.

### Immunization dose assessment in mice

The test vaccine in this study, Vanguard crLyme (Zoetis Petcare), is prepackaged as a 1-mL single-use vaccine (8). The 1-mL dose is formulated for use in dogs. To determine the appropriate volume of vaccine to administer to mice, mice were immunized intraperitoneally with 75, 150, and 275 µL of manufacturer-preformulated crLyme on days 0 and 28. Two weeks after immunization, the mice were euthanized, blood was collected by cheek bleed, and serum was harvested. To determine whether the mice seroconverted, the sera were tested for Abs to CH14 and OspA by single-point ELISA (1:1,000 dilution), as detailed below.

### Immunization of *B. burgdorferi*-infected and -uninfected mice

Two groups of mice (G1 and G2; *n* = 5 mice per group) were needle inoculated with 10^4^ *B. burgdorferi* cells. A third study group (G3) received phosphate buffered saline (PBS; 50 µL). Twenty-seven days later, blood was sampled from all mice, and then mice were administered PBS (G1; 225 µL) or immunized with 225 µL of crLyme (G2 and G3) (Zoetis Petcare). On day 48, blood was collected from all mice by cheek bleed. Serum collected on days 27 and 48 were screened for Abs to DCFL4, VlsE, CH14, and OspA by ELISA (1:1,000 dilution), as detailed below. A summary of the study groups and experimental timeline is presented in Table 1.

**TABLE 1:** Study groups and experimental timeline

Study group	Procedures		
	Day 0	Day 27	Day 48
G1	Infected with 10^4^ *B. burgdorferi* B31; serum collected	Serum collection followed by immunization with PBS: ELISA and immunoblot analyses	Serum collection: ELISA, immunoblot (OspC), and bactericidal assays
G2	Infected with 10^4^ *B. burgdorferi* B31; serum collected	Serum collection followed by immunization crLyme (225 µL): ELISA and immunoblot analyses (OspC)	Serum collection: ELISA, immunoblot (OspC), and bactericidal assays
G3	No treatment	Serum collection followed by immunization with crLyme (225 µL); ELISA and immunoblot analyses (OspC)	Serum collection: ELISA

### SDS-PAGE and immunoblot analyses

Twenty-two recombinant OspC-type proteins (500 ng each) were fractionated by SDS-PAGE using AnyKd precast gels (Bio-Rad) and transferred to polyvinylidene fluoride (PVDF) membranes (Pierce) by semi-dry blotting using a Trans-Blot Turbo apparatus as per the manufacturer’s protocol (Bio-Rad). Non-specific binding sites on the blots were blocked by incubation in Everyblot Blocking buffer (5 min, room temperature; Bio-Rad). Aliquots of serum from each study group were separately pooled and incubated (1 h; room temperature), with the blots at a dilution of 1:1,000 in PBMT (PBS supplemented with 5% non-fat milk and 0.2% Tween 20). The blots were washed three times using PBS-T (PBS with 0.2% Tween 20). Horseradish peroxidase (HRP)-conjugated goat anti-mouse IgG (H/L) (1:40,000; Novus) was added (1 h; room temperature), followed by three washes with PBS-T. IgG binding was detected by chemiluminescence using Clarity Western ECL substrate (Bio-Rad) and imaged with a ChemiDoc Touch (Bio-Rad). Images were cropped for presentation purposes.

### Enzyme-linked immunosorbent assay (ELISA) analyses

ELISA analyses were performed as previously described (26). In brief, the wells of ELISA plates were coated with protein (500 ng/well) using coating buffer (35 mM Na bicarbonate, 15 mM Na carbonate; pH 9.6; overnight; 4°C). Bovine serum albumin served as an immobilized control protein for assessing non-specific binding. Non-specific binding sites were blocked by incubation in PBMT (PBS supplemented with 5% non-fat milk and 0.05% Tween-20). Mouse serum diluted in PBS-T (1:1,000) was added (1 h; room temperature; with shaking), and the plates were washed six times with PBS-T. All ELISA plate wash steps were conducted with an automated plate washer. After washing, HRP-conjugated goat anti-mouse IgG (H/L; Novus Biologicals) was added (1:15,000; 1 h), and the plates were washed as above. 2,2′-Azino-*bis*(3-ethylbenzothiazoline-6-sulfonic acid) substrate (Sigma) was added, and the plates were incubated in the dark (20 min; room temperature). Absorbance was read at 405 nm using an ELx808 plate reader (BioTek).

### Bactericidal antibody assays

The bactericidal activity of serum from each mouse was assessed using *B. burgdorferi* B31, DRI85A, DRI16C, and DRI03C as test strains. All mouse sera were incubated at 56°C for 30 min before use to heat inactivate (HI) endogenous complement. The pooled HI mouse sera (4 µL) from G1, G2, and G3 were combined with 4 µL of late log phase cultures and incubated with guinea pig serum (GPS; 4 µL; CompTech) or HI-GPS. The final volume was brought to 20 µL with BSK-H complete media, and the mixtures were incubated for 18 h at 34°C. Cells in cultivation media with 40% GPS or 20% HI-GPS in preimmune serum (final concentration) served as controls. The average number of live cells in five fields of view was determined by visually counting motile spirochetes using wet mounts and dark-field microscopy. Percent killing was determined for each treatment. All bactericidal assays were performed in triplicate with two technical replicates.

### Statistical analyses

Statistical analyses were conducted using GraphPad Prism version 9.2.0 (GraphPad Software). Ordinary two-way analysis of variances were fit with a full interaction model and significance (*P* < 0.05) calculated by Tukey’s multiple-comparisons test using default settings.

## RESULTS

### Optimization of vaccine and cell inoculum doses

**FIG 1:**
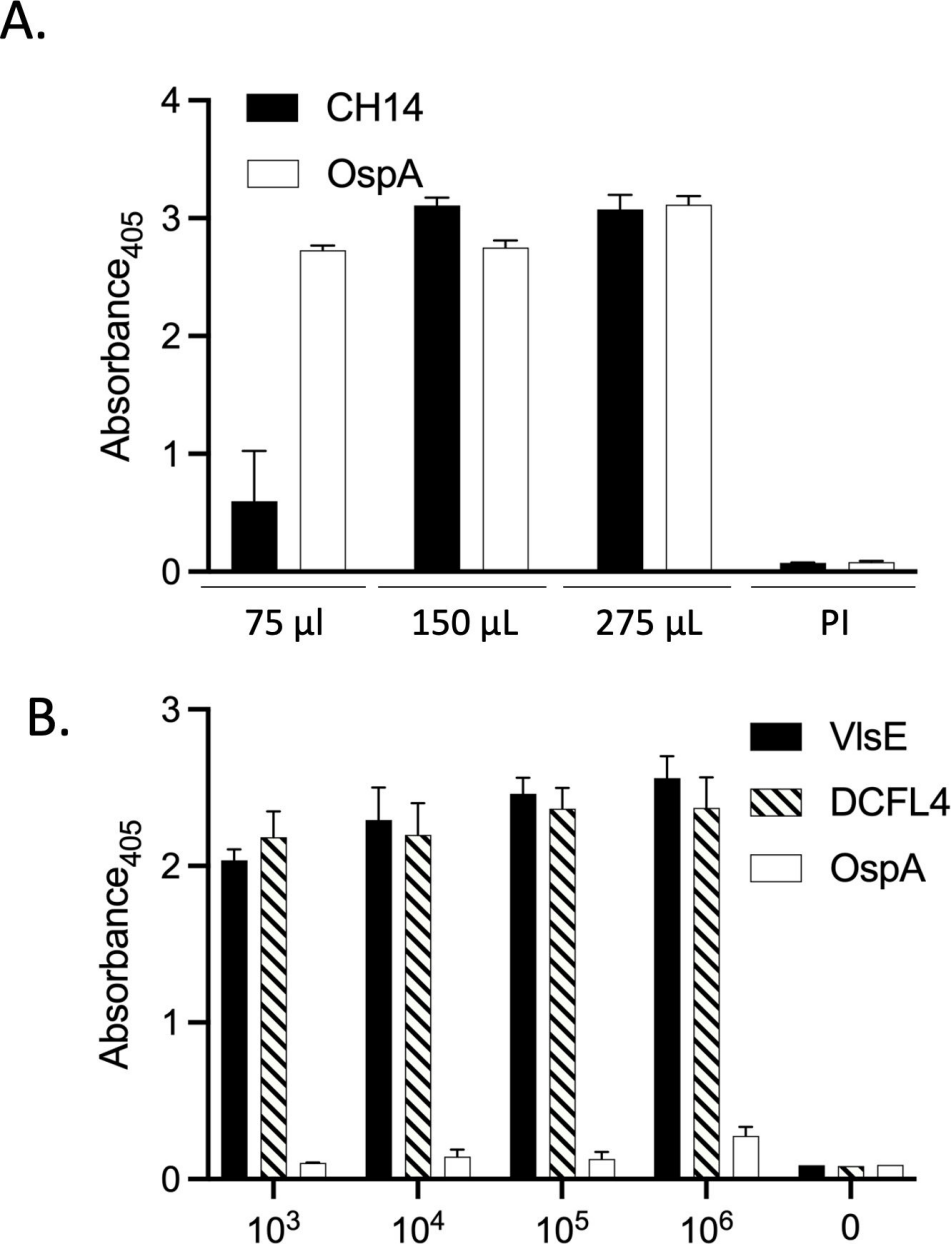
Determination of the optimal vaccine volume and *B. burgdorferi* infectious dose. Increasing volumes of manufacturer-formulated Vanguard crLyme (Zoetis Petcare) were delivered to mice in a two-dose series on days 0 and 28. Fourteen days after the second immunization, serum was harvested, diluted 1:1,000, and screened by enzyme-linked immunosorbent assay (ELISA) for antibodies (Abs) to the vaccine immunogens, CH14 and OspA (A). Preimmune serum served as a negative control. To determine an infectious dose of *B. burgdorferi* that reliably infects mice without eliciting Ab responses to OspA, a protein produced during cultivation but not during infection, increasing numbers of cells ranging from 0 to 10^6^ were administered by subcutaneous needle inoculation. Four weeks later, serum was harvested, diluted 1:1,000, and screened by ELISA for Abs to DCFL4, VlsE (diagnostic antigens), and OspA (B). All assays were done in triplicate, and the average absorbance value for each treatment group was determined. Methods are as detailed in the text. Note that one of five mice that received 10^3^ spirochetes did not seroconvert. The absorbance values for that mouse were not included in the calculations.

Vanguard crLyme is formulated for dogs as a 1-mL dose to be delivered subcutaneously in a prime-boost series 3 weeks apart (7). To conduct this study, we first determined the appropriate vaccine volume for delivery to mice. Mice were immunized with 75, 150, or 275 µL of preformulated vaccine. Ten days after the boost, blood and sera were harvested and screened by ELISA for Abs to CH14 and OspA. Ab levels for both vaccinogens plateaued with vaccine volumes between 150 and 275 µL (Fig. 1A). Based on these data, a vaccine dose of 225 µL was used for immunization in all remaining experiments. The dose of *B. burgdorferi* cells that reliably infect mice without eliciting an Ab response to the bacterial inoculum itself was identified by needle inoculation of mice with increasing numbers of *B. burgdorferi* B31 cells. Sera collected 21 days post-infection were screened by ELISA for Abs to DCFL4 and VlsE. All mice, except one that received 10^3^ spirochetes, were Ab-positive for DCFL4 and CH14 (Fig. 1B), indicating active infection. To determine whether the inoculum elicited Ab responses, the sera were screened for Abs to OspA. Since OspA is produced specifically during cultivation (and in ticks) and not during *Borreliella* residence in mammals, the expectation is that Abs to OspA would only be detected if induced by the inoculum itself (13). None of the mice, including those that received 10^6^ *B. burgdorferi* cells, developed detectable Ab to OspA (Fig. 1B), confirming that the inoculum itself did not trigger Ab production. For all subsequent analyses, mice were infected with 10^4^ spirochetes.

### Antibody responses to immunization in infected and naïve mice

**FIG 2:**
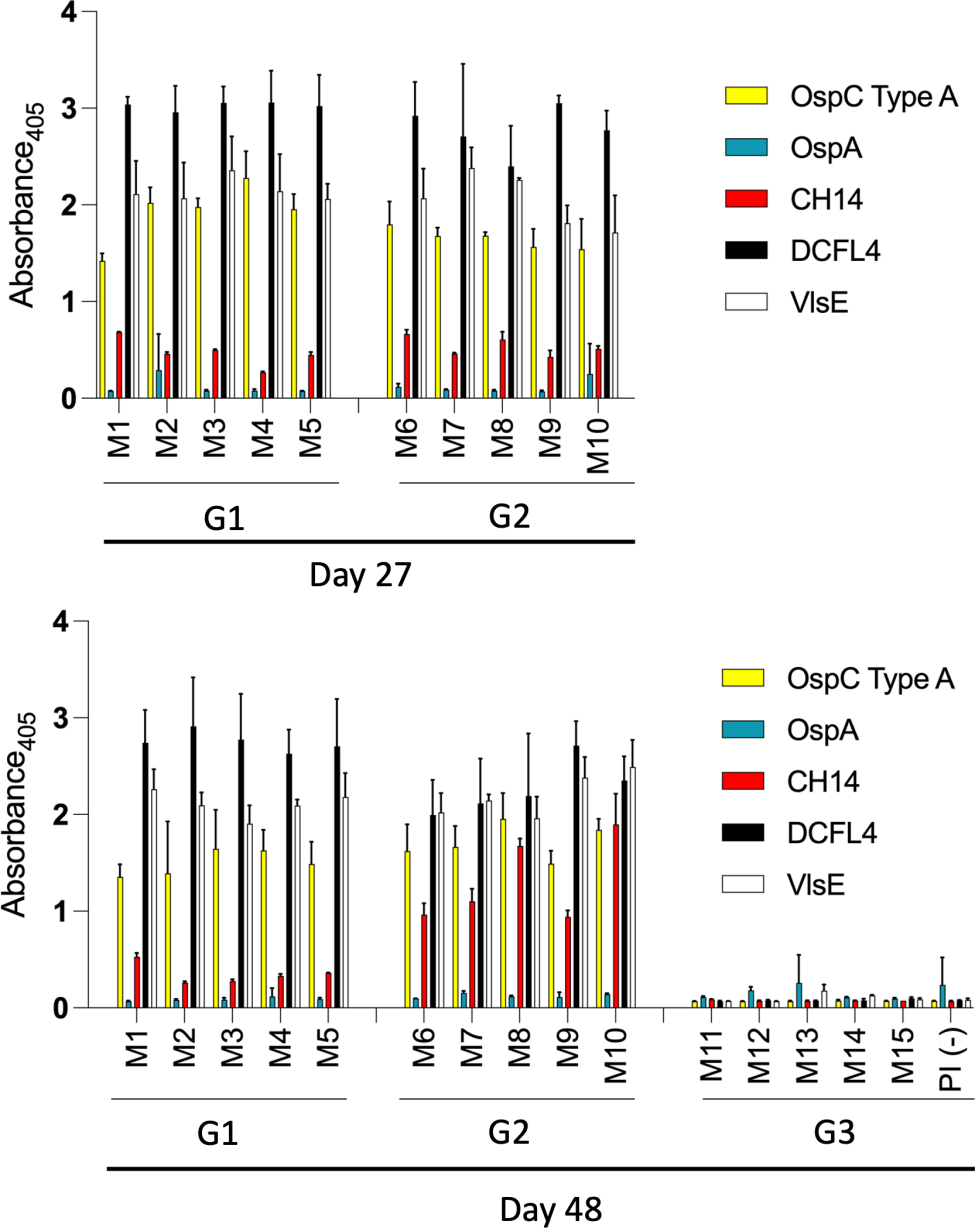
IgG responses of infected and naïve mice to immunization with crLyme. Serum was collected from G1 (infected), G2 (infected but before vaccination), and G3 (before vaccination) mice on day 27. G2 and G3 mice were then vaccinated with 225 µL of manufacturer-preformulated crLyme. Serum was collected from all mice on day 48. Serum samples (diluted 1:1,000) were screened by enzyme-linked immunosorbent assay (ELISA) for antibodies (Abs) to OspC type A (OspC TA), DCFL4, VlsE, CH14, and OspA. All methods are as detailed in the text. Antibody levels of CH14 between groups G1 and G2 were significant (*P* > 0.0001).

Three study groups (G1, G2, and G3) were established as described in Table 1. To determine whether G1 and G2 mice were successfully infected, serum collected on day 27 was screened for Abs to DCFL4, VlsE, CH14, and OspA. All G1 and G2 mice were positive for Abs to DCFL4 and VlsE and negative for OspA, confirming active infection (Fig. 2). Since CH14 is a laboratory-designed recombinant chimeric immunogen (7) that is not naturally produced by *B. burgdorferi*, it can be concluded that Ab binding to CH14 is due to Abs elicited by OspC during infection.

fter collecting blood on day 27, G2 and G3 mice were immunized with crLyme. Sera were collected on day 48 (G1, G2, and G3) and screened for Abs to DCFL4, VlsE, CH14, and OspA (Fig. 2). All G1 and G2 mice were positive for Abs to DCFL4, VlsE, and CH14 and negative for Abs to OspA. The absence of detectable anti-OspA Abs in G1 and G2 mice is consistent with the lack of OspA expression during residence in mammals. The absence of Abs in serum from G3 mice is consistent with the principle that protein subunit vaccines require a prime-boost protocol to elicit detectable Abs. Notably, Ab levels of CH14 in G2 mice were significantly elevated relative to G1 mice (*P* > 0.0001), indicating that immunization of infected mice results in a robust anti-OspC antibody response.

### Immunologically primed mice develop Abs against diverse OspC types after a single vaccine dose

**FIG 3:**
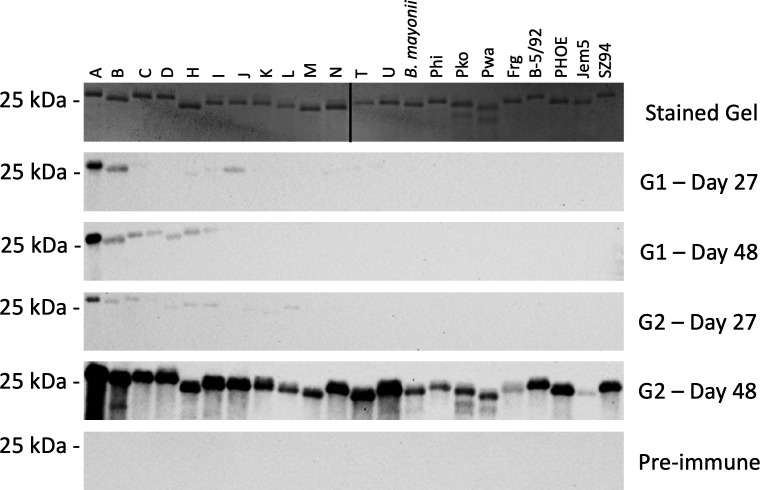
Immunization of *B. burgdorferi* B31-infected mice with crLyme induces antibodies (Abs) that bind to diverse OspC types. Immunoblots of recombinant OspC proteins (type identity indicated above the top panel) were screened with pooled serum (diluted 1:1,000) from study groups G1 and G2 collected on days 27 and 48 (shown on the right). Preimmune pooled serum served as a negative control. The top panel presents a stained SDS-PAGE gel. Methods are as detailed in the text. The study groups and treatments are described in Table 1. The migration position of the 25 kDa molecular weight (MW) marker is indicated on the left. Note that the protein gel was cropped to remove an internal MW marker lane. The location at which the figure was cropped is indicated by a black vertical line.

OspC proteins (*N* = 22) were immunoblotted and screened with serum from G1 and G2 mice. G1 mice developed Abs to OspC that reacted primarily with OspC type A (Fig. 3), consistent with the type specificity of OspC Ab responses in infected mammals (20, 30). Notably, G2 mice (infected and vaccinated) developed Abs that bound to all OspC types tested.

### Measurement of serum bactericidal activity

**FIG 4:**
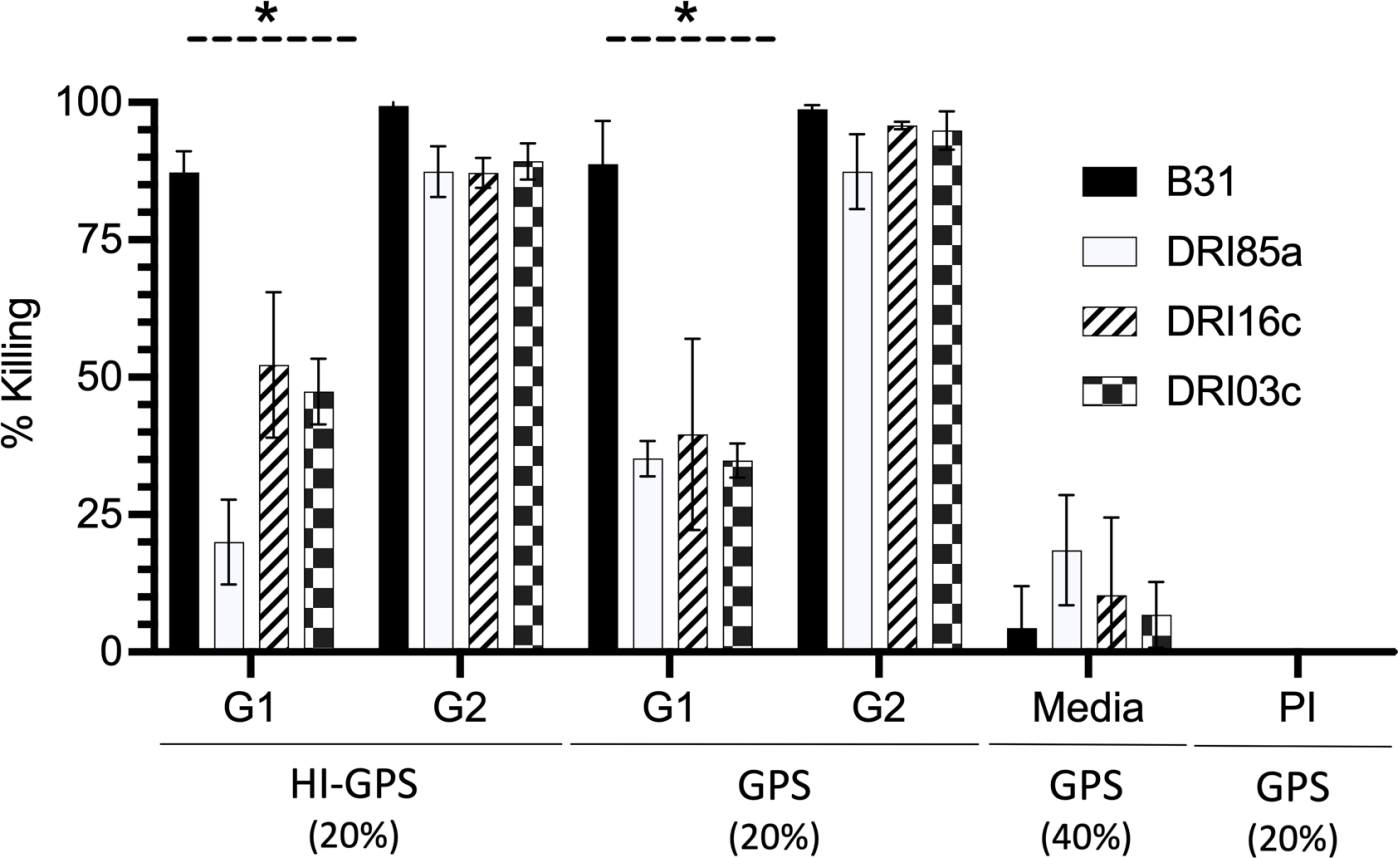
Immunization of *B. burgdorferi* B31-infected mice induces potent bactericidal antibody responses. Day 48 sera from study groups G1 and G2 were individually pooled and assessed for bactericidal activity in the presence of heat-inactivated guinea pig serum (HI-GPS) and GPS as indicated below the graph. Pooled preimmune serum with GPS and media with GPS served as controls. Test strains are indicated in the key. The percentage of cells killed by treatment was determined as detailed in the text.

The serum bactericidal activity from G1 and G2 mice was assessed using diverse *B. burgdorferi* test strains. G1 serum (pooled) killed *B. burgdorferi* B31 cells via an Ab-mediated complement-independent mechanism. The killing of heterologous strains (*B. burgdorferi* DRI85a, DRI16c, and DRI103c) ranged from 20 to 40% (Fig. 4). The addition of GPS (exogenous complement source) did not affect the level of bactericidal activity. G2 serum (pooled) killed more than 86% of the population of homologous and heterologous strains in a complement-independent manner. This finding is consistent with the immunoblots above (Fig. 3), demonstrating that vaccination with crLyme results in a high-level anti-OspC Ab response with broad reactivity.

## DISCUSSION

Vanguard crLyme is a United States Department of Agriculture (USDA)-approved vaccine for the prevention of infection with *B. burgdorferi* in dogs (7). It is comprised of two recombinant proteins: CH14 and OspA. The goal of this study was to determine whether *B. burgdorferi*-infected mice (immunologically primed) develop Abs to diverse OspC variants upon immunization with a single dose of crLyme. A previous case study demonstrated that a *B. burgdorferi* Ab-positive dog mounted a robust Ab response against all 20 OspC variants tested after a single dose of vaccine (31). In that study, a client-owned pregnant Rottweiler presented at a clinic with lameness and other symptoms consistent with Lyme disease. The dog tested positive for Abs to the C6 peptide with a Quant C6 Ab concentration of 237 U/mL. The dog was treated with amoxicillin, and 11 days later, nine healthy pups were delivered. The dam received the first of two doses of crLyme 51 days post parturition. At presentation and before each vaccine dose, blood was collected from the dam, and serum was harvested. The serum samples were screened for Abs to several *Borreliella* proteins, including a panel of diverse OspC proteins and the crLyme immunogens, CH14 and OspA. A single dose of crLyme triggered a broad anti-OspC. Here, we employ a murine Lyme disease vaccination model to advance our understanding of how *B. burgdorferi*-infected or immunologically primed animals may respond to vaccination.

Ab levels of DCFL4, VlsE, 22 OspC variants, CH14, and OspA were measured in G1 (infected), G2 (infected/vaccinated), and G3 (vaccinated) mice. In addition, the bactericidal activity of the sera against the infecting strain (*B. burgdorferi* B31) and heterologous strains (*B. burgdorferi* strains DRI85A*,* DRI16C, and DRI03C) was measured. ELISA analyses of serum collected from mice before immunization on day 27 demonstrated that all infected mice (G1 and G2) mounted high levels of Abs that recognize DCFL4 (chimeric diagnostic antigen), VlsE (derived from strain B31), and OspC (type A). Ab binding to the crLyme immunogen, CH14, but not OspA, was observed. Abs that bound to CH14 were likely elicited by the wild-type OspC type A protein expressed by the infecting strain. To assess the specificity of the OspC Ab response in G1 and G2 mice, 22 OspC proteins were screened by immunoblot. Anti-OspC Abs bound almost exclusively to OspC type A.

After sera were collected on day 27, mice in treatment groups G2 and G3 were immunized with crLyme. Three weeks later (day 48), sera were collected and screened for Abs to the immunogens and antigens described above. G2 mice (infected and immunized) developed significantly higher levels of Abs to CH14 than G1 mice (infected only) (*P* > 0.0001). Mice in the G3 group (single immunization) did not develop IgG to the immunogens or the diagnostic antigens. The lack of a detectable Abs in G3 mice is unsurprising as protein-based subunit vaccines require a prime-boost regiment to elicit strong levels of vaccinal Abs.

An earlier study demonstrated that mice infected with a single strain of *B. burgdorferi* develop short-lived strain-specific immunity (23). Consistent with that, bactericidal assays, an *in vitro* correlate of protection assay, revealed that serum from G1 mice (infected) had potent bactericidal activity against strain B31 but low activity against heterologous strains. In contrast, serum from G2 mice (infected and vaccinated) had broad Ab-mediated-independent killing activity. Immunoblot analyses also demonstrated that anti-OspC Abs from G2 mice bound to diverse OspC proteins, whereas serum from G1 mice did not.

The results of this study, coupled with the case report detailed above, suggest that vaccination of immunologically primed animals with a single dose of crLyme elicits high-titer bactericidal Ab that recognizes diverse strains and OspC types. While care must be taken when extrapolating data obtained in mouse studies to potential outcomes in dogs, the data support the possibility that vaccination of *B. burgdorferi*-infected or Ab-positive dogs can induce anti-OspC Ab responses with potent and broad bactericidal activity.
